# Vocal local versus pharmacological treatments for pain management in tubal ligation procedures in rural Kenya: a non-inferiority trial

**DOI:** 10.1186/1472-6874-14-21

**Published:** 2014-02-04

**Authors:** Sarah C Keogh, Kenzo Fry, Edwin Mbugua, Mark Ayallo, Heidi Quinn, George Otieno, Thoai D Ngo

**Affiliations:** 1 Research, Monitoring, and Evaluation Team, Health System Department, Marie Stopes International, 1 Conway Street, London W1T 6LP, UK; 2 Impact Analysis, Health System Department, Marie Stopes International, London, UK; 3 Research, Monitoring, and Evaluation Team, Marie Stopes International Kenya, Nairobi, Kenya; 4 USAID Support for International Family Planning Organizations Project (SIFPO) – Marie Stopes International, London, UK; 5 Department of Health Management and Informatics, School of Public Health, Kenyatta University, Nairobi, Kenya

**Keywords:** Vocal local, Kenya, Family planning, Pain management, Tubal ligation

## Abstract

**Background:**

Vocal local (VL) is a non-pharmacological pain management technique for gynecological procedures. In Africa, it is usually used in combination with pharmacological analgesics. However, analgesics are associated with side-effects, and can be costly and subject to frequent stock-outs, particularly in remote rural settings. We compared the effectiveness of VL + local anesthesia + analgesics (the standard approach), versus VL + local anesthesia without analgesics, on pain and satisfaction levels for women undergoing tubal ligations in rural Kenya.

**Methods:**

We conducted a site-randomised non-inferiority trial of 884 women receiving TLs from 40 Marie Stopes mobile outreach sites in Kisii and Machakos Districts. Twenty sites provided VL + local anesthesia + analgesics (control), while 20 offered VL + local anesthesia without additional analgesics (intervention). Pain was measured using a validated 11-point Numeric Rating Scale; satisfaction was measured using 11-point scales.

**Results:**

A total of 461 women underwent tubal ligations with VL + local anesthesia, while 423 received tubal ligations with VL + local anesthesia + analgesics. The majority were aged ≥30 years (78%), and had >3 children (99%). In a multivariate analysis, pain during the procedure was not significantly different between the two groups. The pain score after the procedure was significantly lower in the intervention group versus the control group (by 0.40 points; p = 0.041). Satisfaction scores were equally high in both groups; 96% would recommend the procedure to a friend.

**Conclusion:**

VL + local anesthesia is as effective as VL + local anesthesia + analgesics for pain management during tubal ligation in rural Kenya. Avoiding analgesics is associated with numerous benefits including cost savings and fewer issues related to the maintenance, procurement and monitoring of restricted opioid drugs, particularly in remote low-resource settings where these systems are weak.

**Trial registration:**

Pan-African Clinical Trials Registry PACTR201304000495942.

## Background

In rural sub-Saharan Africa, the provision of analgesics during gynecological procedures can be expensive, subjected to frequent stock-outs, and associated with potential side effects. Vocal local (VL) was developed as an alternative to pharmacological approaches to pain management. In these settings, Marie Stopes International (MSI) uses VL and local anesthesia (LA) in place of opioid analgesics, during “mini-laparotomy” ligation. However, amongst other providers in African settings, standard practice for pain management during TL is to administer VL and LA in combination with pharmacological analgesics, under the assumption that VL provides inadequate pain relief without additional analgesics.

VL has been hypothesized to increase client satisfaction by reducing treatment times, giving women a more active role, and emphasizing client-provider relationships. The Vocal Local technique is a continuous process starting from the moment the client enters the clinic, into their consultation, procedure, recovery, through to their discharge from the clinic. VL is based on anxiety reduction, distraction from pain, and avoidance of pain. Anxiety is minimized by having a de-medicalized environment; trained, non-judgmental staff; positive language empowering women to manage their pain; and breathing exercises. Distraction techniques include empathetic continuous conversation that avoids describing the procedure, use of open-ended questions, and structured empathetic attention to help the client manage the pain in the event that she cannot take her attention away from it. Pain is avoided through the use of gentle clinical techniques with non-rigid instruments, and non-use of uterine elevators. However, the effectiveness of VL has not been rigorously evaluated. Evidence shows that a pleasant de-medicalized environment and friendly staff increase client satisfaction [1,2]. Some studies suggest that distraction techniques reduce pain when used in adjunct to analgesics during acute pain and surgical procedures [3-5]; but their effectiveness as a substitute for analgesics has not been assessed. Some evidence suggests that empathetic attention reduces pain, anxiety, need for drugs and procedure time [6], yet other studies showed it hinders patients’ ability to cope with the pain [7]. While one study found that relaxation reduced pain following gynecological surgery [8], a review [9] showed that rhythmic breathing (used in VL) was not effective. Another review evaluating relaxation as the sole analgesic found limited evidence that relaxation was an effective form of pain relief [10].

The existing literature has examined non-pharmacological techniques as a supplement rather than a substitute to analgesics, and as separate components rather than a package (which may alter their effectiveness). Many of these studies had small sample sizes, and most were conducted in urban or hospital settings rather than low-infrastructure rural clinics. We conducted a non-inferiority study to evaluate the effectiveness and acceptability of the VL package as a substitute for pharmacological pain relief during TL in rural settings.

## Methods

A site-randomized controlled non-inferiority study was conducted to compare the effect of VL and local anesthetic (VL + LA) versus VL + LA + analgesics, on pain and satisfaction in women undergoing TL in rural Kenya. The local anesthetic consisted of 2% lignocaine, while the analgesic consisted of 50 mg of Tramadol Hydrochloride (an opioid). The objective was to test whether VL + LA provide at least equivalent pain relief as the standard approach of using VL + LA with pharmacological analgesics. This study was approved by the Ethics Review Committee at MSI and the in-country Ethics Review Committee at Kenyatta National Hospital, Nairobi, Kenya.

The study was conducted from 6^th^ February to 14^th^ July 2012 in 40 MSI mobile outreach clinics in rural Kisii, Nyamira, Machakos, Kitui, Makueni and Kajiado counties, Kenya. MSI delivers TL via mobile outreach teams working with national family planning programs, providing low infrastructure facilities with the equipment and skills necessary for the procedures. Women aged ≥18 opting for TL at participating clinics during the study period were informed about the study’s aims, confidentiality agreements, their right to not participate or to put any questions they wanted, and were asked for their consent to participate. Their response was recorded on the consent form, the interviewer signed next to it and showed the client (clients did not sign themselves as many are illiterate), and each participant was given an information sheet to take home. Clients were then screened for medical eligibility to undergo the procedure, following routine protocol for tubal ligations. Individuals with uncontrolled epilepsy, renal impairment, hypotension, recent head injury and sexually transmitted infections were excluded from the study.

The VL technique was standardized across study sites by including only MSI mobile outreach clinics, where providers received the same training. Clinics were divided into two groups: control sites (using VL + LA + analgesics) and intervention sites (using VL + LA). Half of the clinics (n = 20) located in Kisii, Nyamira and Machakos belonged to the intervention group, while the other 20 clinics in Kitui, Makueni and Kajiado were in the control group. Two outreach teams, consisting of a doctor and a nurse experienced in TL and an assistant specialized in VL, served each study group, and all underwent the same training; having more than one team serving each group ensured that differences between groups were not due to a particular provider’s experience and characteristics. The VL assistants received intensive training in VL when they first started in the mobile outreach TL team, as well as refresher training before the start of the study. All assistants were highly trained and experienced in delivering the technique.

Women attending control sites received VL + LA (consisting of 10ml of 2% lignocaine), coupled with 50 mg of Tramadol Hydrochloride (an opioid analgesic) injected intramuscularly thirty minutes prior to the procedure. VL + LA + analgesics is the standard pain management approach for TL in most African settings outside MSI clinics. In the intervention clinics, women were offered VL and LA without any additional analgesics. Since (in contrast to other providers) the MSI standard is to use VL + LA without analgesics (the “intervention” treatment), the study did not withhold any standard treatment in the intervention clinics, and women were informed that they would not receive an analgesic (following standard practice in MSI clinics). In the “control” clinics, women were offered the MSI treatment (VL + LA) as well as an analgesic, which is the standard package offered by other (non MSI) tubal ligation providers.

After being discharged, all eligible women were invited to complete a face-to-face structured questionnaire with their informed consent. To minimize biases, the interview was administered outside the clinic by non-MSI staff. The main outcomes were pain during the procedure as a whole, pain at the most painful moment of the procedure (which was determined by each woman individually, and could be any time between entering and exiting the procedure room), pain after the procedure, and satisfaction with the procedure. Pain was measured using the 11-point Numeric Rating Scale (NRS), a visual scale from 0 (no pain) to 10 (extreme pain). The NRS has been extensively validated [11,12], including in the context of acute gynecological pain and in low-resource settings [13,14], showing high acceptability [15-17], reliability [15,16], sensitivity [12], response rate [16], and low error rates [17]. Satisfaction was measured using an 11-point numeric scale, Likert-type scales and pre-coded categorical response questions. Secondary outcomes included anxiety during the procedure, experience of side effects, and treatment time.

As this was a non-inferiority study, the sample size was determined based on how much higher the pain score in the intervention group would have to be to show that VL + LA should not be used without analgesics (equivalence margin). Studies using the NRS in combination with physiological measures of pain have shown that the minimum *clinically* relevant difference in pain is around 1.3 [18,19]. The standard deviation (SD) of pain scores during gynecological procedures tends to fall within the range of 3–3.5 using an 11-point scale [20,21]. Assuming a SD of 4 and a design effect of 2 to account for clustering at the clinic level, the sample size required to detect a difference of 1.3 in pain scores between groups was 326 per group, with 90% power at the 5% significance level. This was increased to 500 to allow for a design effect larger than 2.

Analyses were performed in STATA 12 (StataCorp, College Station, TX, USA). Results were adjusted for clustering at the clinic level. After examining crude differences between groups, multivariate linear regression was used to measure the difference in pain and satisfaction scores between groups, controlling for women’s characteristics. Because women had to retrospectively evaluate their pain during the procedure, pain reporting may vary with time elapsed between procedure and interview, so this was controlled for.

## Results

**Figure 1:**
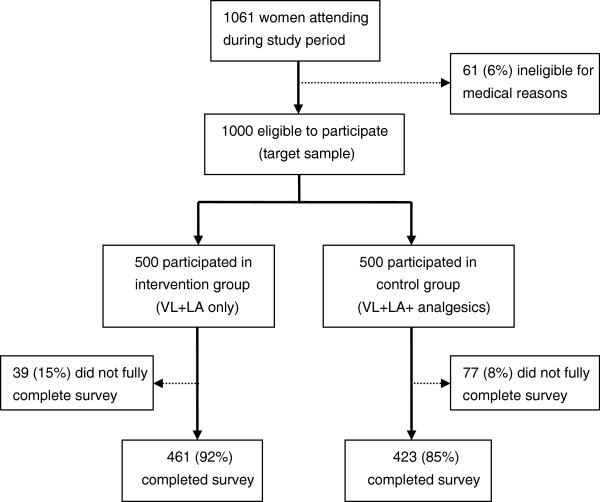
Study cohort profile.

All women attending the clinics for TL during the recruitment period consented to participate in the study; after screening, 61 women were ineligible for medical reasons (Figure 1). Recruitment stopped when the target sample size of 500 in each group was reached. Of 1,000 women, 77 in the control group and 39 in the intervention group did not complete the survey, citing time constraints. In total, 889 women completed interviews: 423 (85%) in the control group and 461 (92%) in the intervention group.

The majority of respondents were over 30 (78%), married (96%) with 3 or more children (99%). There were no differences between the two groups in socio-demographic characteristics or time between the end of the procedure and the interview (~24 minutes) (Table 1).

**Table 1:** Characteristics of women undergoing tubal ligation, by control and intervention groups

	**VL + LA + analgesics (control) N = 423**	**VL + LA only (intervention) N = 461**	**p-value**^**a**^
**Socio-demographic characteristics**	**% (n)**	**% (n)**	
Age group			
*<25*	3.09 (13)	1.74 (8)	
*25-29*	18.53 (78)	20.61 (95)	
*30-34*	41.57 (175)	38.61 (178)	
*35+*	36.82 (155)	39.05 (180)	p = 0.469
Marital status			
*Married*	95.97 (405)	96.53 (445)	
*Not married*	4.03 (17)	3.47 (16)	p = 0.690
Educational level			
*None*	1.90 (8)	2.39 (11)	
*Primary*	62.23 (262)	63.56 (293)	
*Secondary*	35.15 (148)	32.32 (149)	
*College*	0.71 (3)	1.74 (8)	p = 0.607
Area of residence			
*Urban/semi-urban*	12.80 (54)	10.41 (48)	
*Rural*	87.20 (368)	89.59 (413)	p = 0.565
Occupation			
*Unemployed*	13.70 (57)	17.94 (82)	
*Agriculture*	37.50 (156)	40.70 (186)	
*Manual*	47.60 (198)	38.95 (178)	
*Third Sector*	1.20 (5)	2.41 (11)	p = 0.266
Number of births			
*1-2*	1.18 (5)	1.08 (5)	
*3-4*	39.01 (165)	39.26 (181)	
*5-6*	43.50 (184)	46.20 (213)	
*7+*	16.31 (69)	13.45 (62)	p = 0.704
**TL related characteristics**			
Who made decision for TL uptake			
*Respondent*	14.18 (60)	14.10 (65)	
*Partner*	1.18 (5)	1.95 (9)	
*Jointly with partner*	84.16 (356)	83.08 (383)	
*Others (eg family)*	0.47 (2)	0.87 (4)	p = 0.789
Had counselling about TL before			
*No*	46.68 (197)	45.65 (210)	
*Yes*	53.32 (225)	54.35 (250)	p = 0.877
Travel time from home to facility			
*<1 hour*	49.29 (208)	44.69 (206)	
*1-2 hours*	46.92 (198)	52.06 (240)	
*3+ hours*	3.79 (16)	3.25 (15)	p = 0.607
Waiting time at outreach facility			
*<1 hour*	3.79 (16)	3.47 (16)	
*1-2 hours*	31.28 (132)	31.02 (143)	
*3-4 hours*	42.42 (179)	48.81 (225)	
*5+ hours*	22.51 (95)	16.70 (77)	p = 0.466
Anxiety level when entering facility			
*Not at all anxious*	79.62 (336)	83.51 (385)	
*A little bit anxious*	17.06 (72)	13.45 (62)	
*Anxious*	2.61 (11)	1.30 (6)	
*Very anxious*	0.71 (3)	1.74 (8)	p = 0.298

^a^p-value for Chi-square.

Women reported pain levels ranging from 0 to 10 on the three different measures: pain during the procedure, pain at the most painful moment of the procedure, and pain at the time of interview, with medians between 6 and 8. The spread of scores was widest for pain at the time of interview [±SD = 2.11; interquartile range (IQR) = 4], and narrowest for pain at the most painful moment [±SD = 1.73; IQR = 2]. Scores displayed a normal distribution with a slight negative skew (between −0.32 and −0.08) for each of the three measures (Table 2).

**Table 2:** Pain and anxiety experienced by women in control and intervention groups

	**VL + LA + analgesics (control) N = 423**	**VL + LA only (intervention) N = 461**	**p-value**^**a**^
Relaxation when lying waiting (%)			
*Very relaxed*	46.34	40.78	
*Relaxed*	44.21	48.16	
*Not really relaxed*	8.98	9.33	
*Not relaxed at all*	0.47	1.74	p = 0.520
**During procedure**			
Anxiety during procedure (%)			
*Not anxious at all*	35.93	34.06	
*A little bit anxious*	56.74	57.70	
*Anxious*	4.49	5.86	
*Very anxious*	2.84	2.39	p = 0.849
Pain intensity compared to expected (%)			
*Less than expected*	31.44	31.24	
*As expected*	42.32	42.52	
*More than expected*	26.24	26.25	p = 0.995
Moment pain was most intense (%)			
*Cutting/opening of abdomen*	1.18	1.08	
*Hooking/insertion of instrument*	96.93	97.61	
*Closing/stitching*	1.89	1.30	p = 0.801
Percent reporting pain was too much	8.04	6.29	p = 0.573
Percent experiencing any side effect	5.44	3.69	p = 0.376
*Drowsiness*	4.55	0.00	p = 0.440
*Dizziness*	36.36	20.00	p = 0.398
*Nausea/vomiting*	13.64	33.33	p = 0.113
*Pain elsewhere*	27.27	26.67	p = 0.965
*Fast heartbeat*	9.09	0.00	p = 0.257
*Fever*	22.73	33.33	p = 0.443
Mean pain scores (SD)			
*Mean score overall*	6.63 (1.81)	6.46 (1.94)	p = 0.172
*Mean score during most painful moment*	7.00 (1.68)	6.88 (1.77)	p = 0.467
**After procedure**			
Mean pain score at interview (SD)	5.58 (2.14)	5.23 (2.07)	p = 0.091
Percent still experiencing any side effect	23.64	23.43	p = 0.940
*Drowsiness*	9.00	4.63	p = 0.272
*Dizziness*	39.00	27.78	p = 0.230
*Nausea/vomiting*	37.00	33.33	p = 0.672
*Pain elsewhere*	31.00	34.26	p = 0.538
*Fast heartbeat*	0.00	0.93	p = 0.331
*Fever*	15.00	31.48	p = 0.015
*Difficulty breathing*	0.00	0.93	p = 0.308

^a^p-value for Chi-square test for proportions; p-value for t-test for mean pain scores.

Satisfaction scores ranged from 1 to 10 on all four of the measures (Table 3). Over 90% of respondents chose 10 out of 10 on each measure, resulting in strong negative skews between −5 and −7. Over 25% of women expected the visit to be shorter, while 8% felt worse than expected after the procedure.

**Table 3:** Satisfaction measures for women in control and intervention groups

	**VL + LA+ analgesics (control)**	**VL + LA only (intervention)**	**p = value**^**b**^
*Satisfaction scores (SD*^*a*^*)*			
Mean satisfaction with procedure	9.83 (0.76)	9.77 (0.90)	p = 0.484
Mean satisfaction with health provider	9.88 (0.67)	9.84 (0.69)	p = 0.549
Mean satisfaction with care after procedure	9.85 (0.73)	9.87 (0.67)	p = 0.811
Mean satisfaction with length of visit	9.72 (1.10)	9.81 (0.78)	p = 0.329
Length of visit compared to expected (%)			
*Longer*	29.55	26.90	
*About the same*	40.19	38.18	
*Shorter*	30.26	34.92	p = 0.687
Procedure length compared to expected (%)			
*Longer*	4.27	1.97	
*About the same*	8.29	6.55	
*Shorter*	87.44	91.48	p = 0.329
Well-being at time of interview (%)			
*Very well*	19.39	17.79	
*Well*	64.07	70.07	
*Neither well nor unwell*	11.11	9.33	
*Not too well*	5.20	2.60	
*Not well at all*	0.24	0.22	p = 0.512
Well-being at interview compared to expected (%)			
*Better*	41.61	42.95	
*As expected*	50.12	48.81	
*Worse*	8.27	8.24	p = 0.947
Consultation was private (%)	96.69	99.57	p = 0.006
Procedure performed privately (%)	98.58	99.78	p = 0.082
Would have liked more information (%)	85.82	85.90	p = 0.989
Felt comfortable during procedure (%)	98.10	100.00	p = 0.035
Would recommend this clinic to a friend (%)			
*Yes*	95.51	96.10	
*No*	1.18	0.65	
*Unsure*	3.31	3.25	p = 0.743
Mean treatment time in mins (±SD)	6.03 (3.35)	5.44 (2.58)	p = 0.228

^a^SD = standard deviation; ^b^p-value for t-test for difference.

Bivariate analyses showed no significant differences between groups in pain and anxiety (Table 2). The proportion of women reporting side effects during the procedure and at the time of interview did not vary by study group, except for fever at the time of interview, which was more prevalent among women in the intervention group (31% vs. 15%; p = 0.015). The two groups had similar mean satisfaction scores above 9.7, and median scores of 10. A higher proportion of women in the intervention group than the control group reported that their consultation was private (99.6% vs. 96.7%; p = 0.006), and that they felt comfortable during the procedure (100% vs. 98.1%; p = 0.035) (Table 3).

In a multivariate linear regression adjusting for socio-demographic and other personal characteristics related to TL (Table 4), the intervention group had non-significantly lower pain scores than the control group during the procedure as a whole and at the most painful moment. After adjusting for other factors, mean pain at the time of interview was significantly lower in the intervention (VL + LA) group than the control group, by 0.40 points [95% CI: 0.02-0.78].

**Table 4:** **Adjusted**^**a **^**differences in mean scores of pain and satisfaction**

	**Pain during procedure**	***95% CI***	**Pain at most painful moment**	***95% CI***	**Pain after procedure**	***95% CI***	**Satis-faction score**	***95% CI***
Study group								
*Control (ref)*	0.00		0.00		0.00		0.00	
*Intervention*	-0.23	(-0.50-0.03)	-0.17	(-0.42-0.07)	-0.40	(-0.78--0.02)*	-0.06	(-0.18-0.06)
Marital status								
*Married (ref)*	0.00		0.00		0.00		0.00	
*Not married*	-0.04	(-0.87-0.94)	0.18	(-0.47-0.84)	0.80	(-2.05-0.44)	0.18	(-0.05-0.40)
Educational level								
*None*	-0.25	(-1.31-0.82)	-0.15	(-0.95-0.65)	-1.32	(-2.22--0.42)**°**	-0.32	(-0.88-0.24)
*Primary*	0.00		0.00		0.00		0.00	
*Secondary*	-0.04	(-0.27-0.19)	-0.12	(-0.39-0.14)	0.14	(-0.16-0.44)	0.06	(-0.06-0.19)
*College*	0.86	(-0.35-2.08)	0.35	(-0.58-1.29)	0.66	(-0.83-2.15)	0.23	(-0.01-0.47)
Area of residence								
*Urban/semi-urban*	0.00		0.00		0.00		0.00	
*Rural*	0.14	(-0.31-0.58)	-0.04	(-0.37-0.30)	0.29	(-0.14-0.73)	-0.06	(-0.14-0.26)
Occupation								
*Unemployed*	0.65	(0.08-1.22)*	0.84	(0.29-1.41)**°**	0.73	(0.15-1.31)*	-0.23	(-0.44--0.02)*
*Agriculture*	1.23	(0.87-1.60)^+^	1.04	(0.70-1.42)^+^	1.67	(1.23-2.11)^+^	0.18	(-0.07-0.28)^+^
*Manual*	0.00		0.00		0.00		0.00	
*Third sector*	0.03	(-1.45-1.51)	0.84	(-0.09-1.74)	0.49	(-0.73-1.71)	0.01	(-0.34-0.36)
Number of births								
*1-2*	0.54	(-0.28-1.37)	0.35	(-0.27-1.04)	0.82	(-0.35-1.99)	-0.26	(-0.93-0.40)
*3-4*	0.27	(0.01-0.54)*	0.20	(-0.05-0.45)	0.09	(-0.19-0.36)	-0.01	(-0.14-0.11)
*5-6*	0.00		0.00		0.00		0.00	
*7+*	0.34	(-0.04-0.72)	0.20	(-0.16-0.56)	0.31	(-0.09-0.71)	-0.11	(-0.28-0.06)
**TL related characteristics**								
Who made decision for TL uptake								
*Respondent*	0.00		0.00		0.00		0.00	
*Partner*	0.58	(-0.70-1.86)	0.38	(-0.77-1.54)	0.33	(-0.62-1.27)	-0.40	(-1.35-0.34)
*Jointly with partner*	-0.22	(-0.64-0.20)	-0.42	(-0.80--0.03)*	0.14	(-0.32-0.60)	0.08	(-0.13-0.29)
*Others (eg family)*	-0.78	(-2.41-0.86)	-0.20	(-1.92-1.52)	2.86	(1.08-4.64)**°**	0.28	(-0.29-0.85)
Mean time to clinic - increase for each hour increase in time to clinic	0.06	(-0.18-0.31)	0.08	(-0.09-0.26)	-0.05	(-0.31-0.21)	-0.10	(-0.18--0.01)*
Mean time between procedure and interview (for each minute increase)	Not relevant to model		Not relevant to model		-0.01	(-0.02--0.01)^+^	>0.00	(0.00-0.00)*
Anxiety level when entering clinic								
*Not at all anxious*	0.00		0.00		0.00		0.00	
*A little bit anxious*	0.71	(0.30-1.13)^+^	0.62	(-0.31-0.96)^+^	0.37	(-0.03-0.77)	0.01	(-0.15-0.17)
*Anxious*	0.74	(0.12-1.36)*	0.71	(-0.09-1.44)	-0.02	(-0.90-0.85)	0.07	(-0.26-0.40)
*Very anxious*	-0.02	(-1.75-1.70)	-0.25	(-1.79-1.33)	-0.56	(-2.34-1.21)	0.14	(-0.24-0.52)

^+^p≤0.001; **°**0.001 < p≤0.01; *0.01 < p≤0.05.

^a^All differences are adjusted for all other variables in this table.

Other covariates were significantly associated with pain scores in the overall study population: women who were anxious about the procedure experienced greater pain during the procedure and at their most painful moment than those who were not at all anxious. Women with no education reported less pain at the time of interview than those with primary education. Women in manual jobs reported less pain than those who were unemployed or in agriculture, on all three measures.

All women consented to TL voluntarily at the clinics; however, women who reported that it was primarily others (partner/family) who had decided they should have a TL reported far greater pain (~3 points higher) than those who had decided on their own (p = 0.002). Pain decreased with increasing recovery time between procedure and interview (p < 0.001).

The multivariate analysis found no differences in satisfaction scores between study groups. Satisfaction increased significantly with increasing recovery time between procedure and interview (p = 0.039); and women who had travelled further to the clinic reported lower satisfaction (p = 0.034).

## Discussion

In this non-inferiority study, we showed that VL + LA was as effective on its own as with pharmacological analgesics in managing pain during TL in rural Kenya. After adjusting for other factors, pain during the procedure was not significantly different between the two groups, while pain at the time of interview was significantly lower in the group that did not receive analgesics (intervention). This might be explained by the analgesics wearing off in the control group by the time of the interview (leading to increased pain). The analgesic did not appear to reduce pain even at the most painful moment when administered in addition to VL.

Although women’s characteristics did not differ significantly between intervention and control groups, they were nonetheless adjusted for in the multivariate models. There may however be differences between groups that were not measured and adjusted for (i.e. at the county level, since allocation was by county). With regards to outcome measures, the NRS for pain has been validated in some African settings, but not specifically in Kenya. Kenyan women may have a different understanding of pain relative to women in Western settings (where the scale has mainly been used). Pain scores displayed a normal distribution, suggesting the scale was well understood by respondents.

In contrast, the satisfaction scale exhibited a strong negative skew due to heavy rating of the high scores, questioning its validity. Clients’ satisfaction with health services has been a difficult concept to measure [22], and this may be exacerbated in settings where strong hierarchical relationships prevail between patient and provider. We minimized this issue by using non-MSI interviewers. Different scales should be developed to obtain more sensitive satisfaction measures. The intervention group’s greater satisfaction on certain aspects of their experience (private consultation, comfort during the procedure) calls for further investigation into the implementation of VL. Ensuring that consultations are private and that women feel comfortable are intrinsic components of VL. However, VL should have been implemented comparably in both groups, and the use of analgesics should not have affected these outcomes.

Previous studies [3-5,8,23,24] showed that relaxation and distraction techniques help reduce pain during surgical procedures. This is the first study to evaluate VL as a package and a substitute for pharmacological analgesics in a rural African mobile outreach setting. Going beyond findings from hospital settings, this study provides evidence that VL is as effective on its own as with analgesics, specifically in rural low-resource facilities where replacing analgesics with non pharmacological treatments could lead to significant cost savings and more efficient services. The effectiveness of VL is dependent on high quality provider training: providers had been using VL daily for several years with regular refresher training, and it is uncertain what effect the intervention would have had on pain had providers been less experienced. Further research should examine how the use of VL without analgesics can be standardized and rolled out beyond MSI mobile clinics to other healthcare settings and service delivery models.

The potential benefits of using VL without additional analgesics are numerous. It can reduce the risk of medical error and side effects associated with opioid drugs. Drowsiness from analgesics can lead to longer clinic stays, when many women may prefer a shorter visit. The VL + LA approach would eliminate issues related to the maintenance, procurement and monitoring of restricted opioid drugs, particularly in low-resource settings where such systems are weak. Drug stock-outs in remote settings can delay the procedure or prevent women from having it, which could result in unwanted pregnancies. Employing the low-cost VL technique could lead to substantial savings in settings like Kenya where resources are scarce. If cost-effective, VL could potentially be used as a substitute for analgesics during other ob/gyn procedures, such as caesarean sections. Policy recommendations regarding the wider adoption of VL without analgesics for TL and other procedures should be based on the cost-effectiveness of each approach, using a wider health systems approach.

Caution should be used in generalizing the results of this study to other rural African settings, larger health facilities and urban areas. Women using mobile outreach clinics for TL may exhibit different characteristics to those seeking services at formal health facilities. The VL + LA approach might be well suited to certain populations, while it may be inferior to pharmacological pain relief in other populations. Women in this study did display similar characteristics to those seeking TLs in other healthcare settings in Kenya [25]. However, it is possible that women in other settings might have different expectations regarding pain management, which may in turn impact their pain tolerance. For example, in certain formal health facilities in Kenya, and in health facilities in other countries, standard practice for tubal ligation may involve an analgesic local anesthetic, and it may be unimaginable for women to go without it. Thus, women’s expectations regarding the treatment they will receive and the amount of pain they will endure may affect the level of pain they experience. For this reason, we must be cautious in generalizing these results to other settings where anesthesia is the norm and expectation for tubal ligation.

## Conclusion

This study is the first to evaluate the VL pain management technique as a package for use as a substitute for pharmacological analgesics in gynecological procedures. The findings provide strong evidence that VL + LA is as effective on its own as with additional analgesics for mobile outreach TL services in rural Kenya. Using VL + LA could lead to significant cost savings and more efficient services, particularly in rural low-resource settings.

## Competing interests

The authors declare that they have no competing interest.

## Authors’ contributions

SK developed the protocol and study design and led the data analysis of the manuscript under the guidance of KRF and TDN. SK and TDN co-wrote the manuscript. EM, MA, GO were responsible for the coordination and implementation of the study in Kenya, and participated in data collection, management, and contributed to the data analysis and writing of the manuscript. HQ and KRF participated in the development of the protocol, the implementation of the study and the writing of the report. TDN was the principal investigator who conceptualized the study, and was responsible for the overall supervision of the study and participated in the implementation of study, data analysis, and the writing of the manuscript. All authors had access to the data, commented on subsequent drafts, and approved the final submitted version. All authors read and approved the final manuscript.

## Pre-publication history

The pre-publication history for this paper can be accessed here:

http://www.biomedcentral.com/1472-6874/14/21/prepub
